# Access to appropriate health care for non-English speaking migrant families with a newborn/young child: a systematic scoping literature review

**DOI:** 10.1186/s12913-020-05157-x

**Published:** 2020-04-15

**Authors:** Louise Dougherty, Jane Lloyd, Elizabeth Harris, Paula Caffrey, Mark Harris

**Affiliations:** 1 Health Equity Research and Development Unit, a unit of Clinical Services Integration and Population Health, Sydney Local Health District and a research hub of the Centre for Primary Health Care and Equity, University of New South Wales, Kensington, Australia; 2 Centre for Primary Health Care and Equity, University of New South Wales, Kensington, Australia; 3 Community Health, Sydney Local Health District, Camperdown, Australia

**Keywords:** Health equity, Migration, Maternal and child health, Postnatal care, Access to health care, Emigration and immigration, Migrant mothers

## Abstract

**Background:**

Recently arrived culturally and linguistically diverse migrant mothers in Western Industrialised Nations are less likely to access health care and are more likely to report negative healthcare experiences than more established migrant or non-migrant populations. This is particularly an issue in Australia where nearly half of all Australians were born overseas or have at least one parent born overseas.

**Methods:**

A systematic scoping review was conducted to identify a) the main enablers and barriers to accessing appropriate health care for migrant families with a new baby/young child who speak a language other than English, and b) the effectiveness of interventions that have been tested to improve access to appropriate health care for this group. Three academic databases (CINAHL, Medline and ProQuest) were searched, with additional publications identified through expert knowledge and networks. Data was extracted and analysed according to the Access framework, which conceptualises access to health care as being generated by the interaction of dimensions of accessibility of services (supply side) and abilities of potential users (demand side).

**Results:**

A total of 1964 records were screened for eligibility, with nine of these included in the review. Seven studies only described barriers and enablers to health care access, one study reported on an evaluation of an intervention and one study described the barriers and enablers and the evaluation of an intervention. This review identified that the most significant barriers occurred on the supply side, within the ‘appropriateness’ domain. Overall, the most frequently cited barrier was a lack of cultural sensitivity/understanding of different cultural practices (five studies). The most significant enablers also occurred on the supply side, but within the ‘acceptability’ domain. The most frequently cited enabler was cultural sensitivity and understanding.

**Conclusions:**

There is a dearth of evaluated interventions in the peer reviewed literature to improve appropriate access to postnatal care for migrant families who speak a language other than English. The literature focuses on identifying barriers and enablers to access to healthcare for this population group. Interventions which aim to address barriers within the ‘appropriateness’ dimension may have the greatest impact on access.

## Background

Migration can create or increase vulnerability to ill health, due to a range of factors such as low socioeconomic status, uncertainty about healthcare rights, institutional barriers, stress, and language and cultural differences [1]. This vulnerability can be particularly pronounced during the period surrounding new motherhood, with recently arrived, culturally and linguistically diverse migrant mothers experiencing lower levels of access to health care and poorer birth outcomes than non-immigrants or English-speaking immigrants [2], as well as being more likely to report negative experiences across antenatal, intrapartum and postnatal care [3]. A review of maternal health care inequalities for migrants in the World Health Organization European Region identified that migrant women have less access to family planning and contraception in the preconception period and a higher incidence of poorer outcomes from pregnancy such as induced abortion, caesarean or instrumental delivery or complications [1]. In the postnatal period, migrant women have been reported to experience higher rates of postpartum depression and higher risk of intimate partner violence [4]. Difficulties in the postnatal period may be compounded by the fact that migrant mothers experience a “double transition”, having to adjust to life in a new country as well as to motherhood [5]. This “double transition” may also be relevant to women who have experienced motherhood previously but for whom it is their first baby in a new country.

Access to health care in the postnatal period is important for both mothers and their children. The early years and parenthood represent an ideal stage to intervene to improve access to health care as the early years of a child’s life lay critical foundations for the entire life course, including education and long-term health outcomes [6, 7]. Intervening at this time can prevent lifelong health inequities, particularly the onset of chronic disease. Investing in the early years has one of the greatest potentials to reduce health inequities within a generation [7]. Intensive efforts to promote early childhood development can be seen internationally [7–9] and nationally [10].

To inform the development of a program to increase migrant parents’ access to health care, a scoping systematic literature review was conducted to understand the enablers and barriers to accessing care that have been identified for this population group, as well as the types of interventions that have previously been conducted. While reviews have been conducted previously to identify enablers and barriers to healthcare access for migrant groups, we believe that this is the first review to focus on the postnatal period and to examine these in the context of the Access framework. This provides important insights into which particular aspects of access might be most beneficial to focus an intervention on. This publication reports on the findings of this review.

## Methods

This scoping systematic literature review set out to answer the following questions:

1. What are the main enablers and barriers to accessing appropriate health care for migrant families with a new baby/young child who speak a language other than English?
2. What interventions have been tested to improve access to appropriate health care for migrant families with a new baby/young child who speak a language other than English, and were they successful?

The scoping review was guided by the methodology proposed by Arksey and O’Malley [11]. However, we added a quality appraisal step to this methodology as the quality of studies was used to inform our discussion of whether the interventions were successful.

### Definitions

While there is no formal legal definition of the term ‘migrant’, there is general consensus that in the international context, the term refers to someone who changes his or her country of usual residence, regardless of reason or legal status [12]. The definition of ‘refugee’ is formally outlined in international law, and refers to persons displaced from their country of origin due to violence, persecution or other threats [12]. This review focuses on both migrant and refugee populations, however, for simplicity, the term ‘migrant’ is used throughout to refer to both groups.

### Study selection

Searches were conducted in three academic databases (CINAHL, Medline and ProQuest). The search terms were adapted for each database and included terms for the population and setting of interest. The search was limited to articles published in English from 2003 onwards. An example of the Medline search strategy for the initial search is shown in Table 1.

**Table 1:** Medline search strategy *(executed August 27, 2018)*

#	Search terms
1	Transients and migrants
2	Refugees
3	Postnatal care
4	Infant care
5	Maternal health services
6	1 or 2
7	3 or 4 or 5
8	6 and 7
9	Limit 8 to (English language and yr = 2003-current)

A second search with additional terms relating to child care was run in the same databases. An example of the Medline search strategy for the second search is shown in Table 2.

**Table 2:** Second Medline search strategy *(executed August 27, 2018)*

#	Search terms
1	Transients and migrants
2	Refugees
3	Postnatal care
4	Infant care
5	Child care
6	Child health services
7	Maternal health services
8	1 or 2
9	3 or 4 or 5 or 6 or 7
10	8 and 9
11	Limit 10 to (English language and yr = 2003-current)

Additional publications were identified through an iterative approach. Identified websites of interest were searched for relevant project or program evaluations. Additional publications were sourced through approaching key subject matter experts, in line with recommendations from Greenhalgh et al. (2005) [13] that relying on a formal protocol-driven search strategy alone may result in missing important evidence, with informal approaches important to increase the yield and efficiency of a search.

Inclusion criteria were developed based on the aims and scope of the review. The review was restricted to empirical studies published in 2003 onwards, from an OECD country, which **either** described barriers or enablers to accessing appropriate health care for migrant families with a new baby/young child who speak a language other than English, **and/or** evaluated an intervention supporting appropriate access to health care for this population group. The time frame of 2003 onwards was chosen as it was thought that the most relevant information would be contained in studies published within the last 15 years.

A comprehensive, fit-for-purpose data extraction template was designed and piloted by the research team. Data extraction was conducted by an independent researcher. Double data extraction was conducted on a 20% sample (two articles) (by LD and the independent researcher) at the beginning of the data extraction process to ensure that the independent researcher extracted all relevant information and reported it in a consistent manner.

### Quality appraisal

Included studies underwent quality assessment using tools specific to the study type. For the qualitative studies, the Joanna Briggs Institute Critical Appraisal Checklist for Qualitative Research was used [14]. For the quasi-experimental studies, the Joanna Briggs Institute Critical Appraisal Checklist for Quasi-Experimental Studies was used [15]. Quality assessment was conducted independently by two reviewers (LD and an independent researcher not involved in this research project). Any disagreements were resolved through discussion, until the reviewers’ agreed on all scores.

### Data analysis

**Fig. 1:**
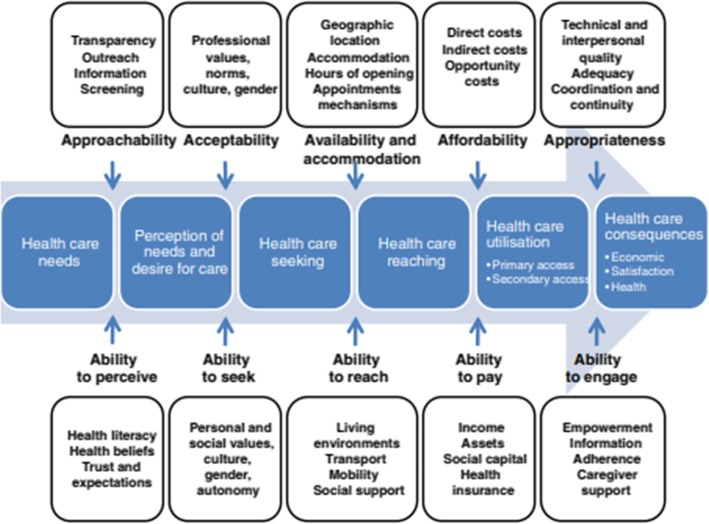
The Access framework [16]

For research question 1, the barriers or enablers identified in the studies were grouped according to the dimensions of the Access framework, which were developed by Levesque et al. [16]. They were grouped in this way in order to determine where the main barriers and enablers exist along the path to obtaining health care. The Access framework posits that there are five dimensions of accessibility of services (supply side) which interact with five abilities of potential users (demand side) to generate access to health care [16] (Fig. 1- supply side dimensions at the top of the figure and demand side abilities at the bottom of the figure). According to the Access model, the five user (demand) dimensions influencing access are ‘Ability to perceive’, ‘Ability to seek’, ‘Ability to reach’, ‘Ability to pay’, and ‘Ability to engage’ [16]. The five service (supply) dimensions are ‘Approachability’, ‘Acceptability’, ‘Availability and accommodation’, ‘Affordability’ and ‘Appropriateness’ [16]. Examples of the factors encapsulated by each dimension are shown in Fig. 1 below.

For research question 2, the components of the intervention activities and the effectiveness of the interventions were described.

## Results

**Fig. 2:**
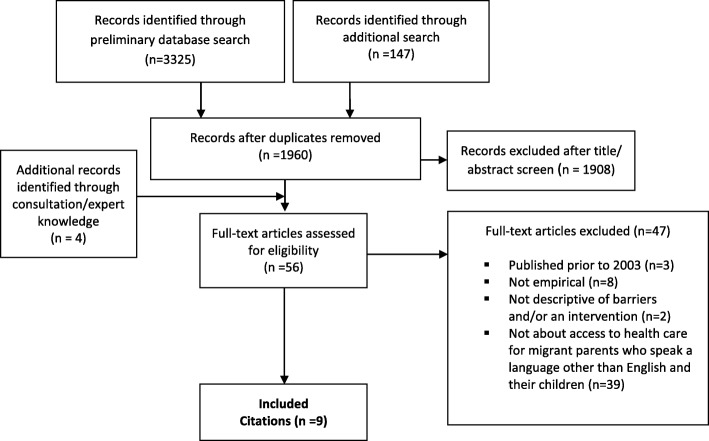
PRISMA flowchart

A total of 1964 citations from the three academic databases were screened for eligibility. Of these, 56 citations underwent full text review. The literature review search process identified nine articles of relevance. Five of these articles were identified through the database search and four were identified through consultation/expert knowledge. No relevant citations were identified through the search of identified websites. The full study selection process is depicted in the PRISMA flowchart below (Fig. 2).

### Descriptive overview

Some descriptive characteristics of the included studies are shown in Table 3 below. The majority of the studies described barriers to health care access, rather than evaluating specific interventions. The studies were mainly qualitative (7 studies), with only two quasi-experimental studies. The studies could be broadly categorized into two focus areas: oral health (of babies and young children) (3 studies), or maternity and postnatal/postnatal alone (6 studies). Two-thirds of the studies identified were Australian and thus applicable to our study setting. Studies included a wide range of ethnicities, with the most common being Afghani (3 studies). The studies mostly had a small number of participants. The studies contained information from a variety of perspectives, including migrant/refugee women, migrant/refugee men, health professionals and voluntary workers. However, the majority of the focus was on the experience of migrant/refugee women (7 studies). More detailed information about each study is shown in Table 4, which contains a summary of each of the included studies.

**Table 3:** Characteristics of the included studies

Study characteristic		No of studies
**Study focus**^**a**^	Description of barriers to health care access	8
	Description of enablers to health care access	8
	Evaluation of an intervention	2
**Health care service focus**	Maternity and postnatal or just postnatal	6
	Oral health of babies/young children	3
**Setting**	Australia	6
	Canada	2
	England	1
**Design**	Qualitative	7
	Quasi-experimental	2
**Target population**	Migrants	3
	Refugees	3
	Mixed	3
**Target ethnicity**^**a**^	Afghani	3
	Chinese	2
	Iraqi	2
	Lebanese	2
	Pakistani	2
	Vietnamese	1
	African	1
	Middle Eastern^b^	1
	Not specified or eligibility not restricted to particular language groups	2
**Number of participants**	< 20	1
	21–60	4
	61–100	2
	100+	2
**Type of participant**^**a**^	Migrant/refugee women	7
	Migrant/refugee men	3
	Health professionals	2
	Voluntary workers	1

^**a**^not mutually exclusive categories

^b^Includes Lebanese participants but they are not the sole focus of the study with Middle Eastern participants, so this study has not been counted separately in the Lebanese category

**Table 4:** Summary of included studies

Author/Year	Country	Aim	Study design	Target population	Health care service focus	Perspective	Number of participants/gender	Quality appraisal score
**Descriptive studies of enablers/barriers to health care access**								
Balaam et al. 2016 [17]	England, UK	To explore the experiences of voluntary sector workers supporting asylum seeking and refugee women during pregnancy and early motherhood	Qualitative	Pregnant refugees and asylum seekers (but authors interviewed workers who work directly with this client group instead due to difficulty in accessing the women directly)	Maternity and postnatal services	Voluntary workers	19 individuals (3 focus groups and 1 interview)- gender not specified	7/10
Chu et al. 2005 [18]	Brisbane, Australia	To examine the postnatal experience and support needs of Chinese migrant women in Brisbane	Qualitative	Chinese migrant women in Australia	Postnatal services	Patients (women)	55 women (face-to-face and telephone interviews); field groups to selected community organisations and focus group discussions (participants not specified)	8/10
Gagnon et al. 2010 [19]	Montreal, Canada	To explore the inhibitors and facilitators for migrant women who have recently given birth following through on referrals made in the community by nurses for additional care for their baby and/or themselves	Qualitative	Women with migration histories who have recently given birth	Postnatal services	Patients (women)	25/75 women (group and individual interviews)	7/10
Renzaho et al. 2013 [20]	Dandenong, Australia	To explore the views and perceptions of migrant women in Dandenong, Australia about sociocultural barriers and health needs during pregnancy and in the postnatal period, and to identify potential solutions to address such barriers	Qualitative	Migrant lactating mothers, with at least one child aged < 3 years (Afghani, African, Chinese and Middle Eastern)	Maternity and postnatal services	Patients (women)	5 focus group discussions with 35 migrant mothers	8/10
Riggs et al. 2014 [21]	Melbourne, Australia	To explore the experiences of dental service use from the perspective of migrant mothers	Qualitative	Migrant mothers living in Melbourne from Iraq, Lebanon and Pakistan	Oral health	Patients (women)	11 focus groups and interviews with 115 women	8/10
Riggs et al. 2016 ^b^ [22]	Melbourne, Australia	To explore the experiences of Afghan men of refugee background having a baby in Melbourne, and the reflections of health professionals about the role of men in maternity and early childhood care	Qualitative	Afghan men of refugee background who had had a baby in Melbourne in the previous month	Maternity and postnatal services	Patients (men) and health professionals	14 Afghan men (interviews); 34 health professionals (interviews and focus groups)	9/10
Yelland et al.^b^ 2014 [23]	Melbourne, Australia	To explore the responsiveness of health services to the social and mental health of Afghan women and men who had recently had a baby	Qualitative	Afghan women and men who had recently had a baby in Melbourne	Maternity and postnatal services	Patients (women and men) and health professionals	30 interviews with Afghan women and men; interviews and focus groups with 34 health professionals; consultation with 100 members of the Afghan community	8/10
**Studies which evaluate an intervention**								
Gibbs et al. 2015 [24]^a^	Melbourne, Australia	To establish and evaluate a model for child oral health promotion for families with migrant backgrounds	Program evaluation (pre/post) with comparison group	Families with 1–4-year-old children, from Iraqi, Lebanese or Pakistani backgrounds)	Oral health	N/A	521 families (691 children) at baseline; 275 families (365 children) at follow up (53%)	9/9
Harrison et al. 2003 [25]	British Columbia, Canada	To design, implement and evaluate an oral health promotion program for Vietnamese pre-school children in Canada	Program evaluation (pre/post) with comparison group	Vietnamese mothers with children under 5 years of age living in Canada	Oral health	N/A	112 mothers at baseline (who had more than one counselling session), 66/112 (59%) at follow-up	5/9

^a^Gibbs study also reports on barriers

^b^ Related papers: one focuses on migrant women and men; the other focuses solely on migrant men

### Quality appraisal

The qualitative studies were all deemed to be of reasonable quality, with all seven qualitative studies scoring 7/10 or higher using the Joanna Briggs Qualitative assessment questions [14]. The scores for the two intervention studies were variable, with one receiving a score of 9/9 and the other receiving a score of 5/9 using the Joanna Briggs Quasi-experimental assessment questions [15]. The study scoring 5/9 was included in the review given the extremely small number of intervention studies identified, although its limitations are discussed in the results section.

### Research question 1: what are the main barriers and enablers to accessing appropriate health care for migrant families with a new baby/young child who speak a language other than English?

Of the nine included studies, eight contained information on barriers to accessing health care for migrant families, and eight contained information on enablers to health care access for this population group. The barriers and enablers mentioned within the studies were identified by a range of individuals, including migrant men, migrant women, health workers and voluntary sector workers.

#### Barriers

Barriers were identified for both the demand and supply side of the Access framework. The distribution of barriers was mostly even across the demand and supply sides (14 compared to 17, respectively). In general, migrant women identified barriers on the ‘demand’ side of the Access model. ‘Supply’ side barriers were more widely recognised by all types of participants.

The most common demand side dimensions where barriers were identified were the first three dimensions- ‘ability to perceive’, ‘ability to seek’ and ‘ability to reach’ (with four barriers identified for each of these three dimensions). Within all of the demand side dimensions, the most commonly identified specific barriers were language difficulties (mentioned in four studies) and loneliness/lack of social support (three studies).

The most common supply side dimension where barriers were identified was ‘Appropriateness’, with eight barriers identified for this dimension. Within all of the supply side dimensions, the most commonly identified specific barriers were lack of cultural sensitivity/understanding of cultural practice differences (mentioned in five studies) and difficulty accessing interpreters (three studies).

A full list of the barriers identified within each of the access dimensions is shown below in Table 5.

**Table 5:** Barriers identified within the studies, arranged within the Access dimensions

Access dimension	Barrier (citation)	Perspective of study			
		Migrant/ refugee women	Migrant/ refugee men	Health professionals	Voluntary workers
**DEMAND SIDE**					
Ability to perceive	Women unfamiliar with the concept of maternity care [17, 18]	✓			✓
	Perceived inappropriate referrals [19]	✓			
	Confusion about how to navigate ‘systems of care’ [20, 21]	✓			
	Previous negative experiences with health care encounters [21]	✓			
Ability to seek	Stress and competing priorities [18]	✓			
	Language difficulties [18–20]	✓			
	Family conflicts [18]	✓			
	Difficulty making appointments [19–21]	✓			
Ability to reach	Transport [18, 19]	✓			
	Loneliness/social isolation/lack of support [18–20]	✓			
	Childcare difficulties [19]	✓			
	Dependence on husband (for transport, interpretation and/or finances) [19, 23]	✓	✓	✓	
Ability to pay	Unstable income or lack of suitable work or employment opportunities [18, 22]	✓	✓	✓	
	Prioritising children’s education [18]	✓			
Ability to engage	N/A				
**SUPPLY SIDE**					
Approachability	Lack of information and resources available for women and families [18, 23]	✓	✓	✓	
	Outdated professional lists [19]	✓			
Acceptability	Lack of cultural sensitivity/understanding of different cultural practices [17–19, 22, 23]	✓	✓	✓	✓
	Negative attitudes [17]				✓
Availability and accommodation	Interpreter services [18, 21, 23]	✓	✓	✓	
	Waiting lists [21]	✓			
	Lack of flexibility [19]	✓			
	Complicated phone systems [19]	✓			
Affordability	Confusion about cost of services/eligibility [21]	✓			
Appropriateness	Poor communication [17]				✓
	Stress of caring role [17]				✓
	Lack of continuity [17]				✓
	Transience of refugee and asylum-seeking women [17]				✓
	Lack of information and training provided to health professionals [17]				✓
	Challenges of multi-agency practice [17]				✓
	Disagreements about patient management [20]	✓			
	Short consultations or lack of continuity [23]	✓	✓	✓	

#### Enablers

Enablers were identified for both the demand and supply side of the Access framework. A greater number of enablers were identified for the supply side than the demand side (12 compared to three, respectively). The identification of enablers was spread reasonably evenly across the different participant groups.

The most common demand side dimension where enablers were identified was ‘ability to reach’ (two enablers identified within this dimension). Within all of the demand side dimensions, only three enablers were identified in total (early receipt of information; family and social support; and community-based services close to home). These were only identified by one study.

The most common supply side dimension where enablers were identified was ‘acceptability’ (four enablers identified within this dimension). Within all of the supply side dimensions, the most commonly identified specific enablers were cultural sensitivity and understanding (mentioned in six studies) and building trusting, empathetic and ongoing relationships (four studies).

A full list of the enablers identified within each of the Access dimensions is shown below in Table 6.

**Table 6:** Enablers identified within the studies, arranged within the Access dimensions

Access dimension	Enabler (citation)	Perspective of study			
		Migrant/ refugee women	Migrant/ refugee men	Health professionals	Voluntary workers
**DEMAND SIDE**					
Ability to perceive	Early receipt of information [19]				✓
Ability to seek	N/A				
Ability to reach	Family and social support [20]	✓			
	Community-based services close to home [23]	✓	✓	✓	
Ability to pay	N/A				
Ability to engage	N/A				
**SUPPLY SIDE**					
Approachability	Encourage community and social support [17, 18]	✓			✓
	Accessible information on health [18]		✓		
Acceptability	Cultural sensitivity and understanding [17–19, 21, 23, 24]	✓	✓	✓	✓
	Involve fathers in care [22]		✓	✓	
	Culturally appropriate services [18]	✓			
	Appropriate referral pathways [19]	✓			
Availability and accommodation	Hold clinics in a familiar, trusted location [17]				✓
	Develop holistic clinics [17]				✓
	Interpreters [21]	✓			
Affordability	N/A				
Appropriateness	Building trusting, empathetic and ongoing relationships [17, 19, 22, 23]	✓	✓	✓	✓
	Thinking ‘outside the box’ [17, 20]	✓			✓
	Longer appointment times [23]	✓	✓	✓	

### Research question 2: what interventions have been tested to improve access to appropriate health care for migrant families with a new baby/young child who speak a language other than English, and were they successful?

The scoping review only identified two studies which tested an intervention aimed at improving access to appropriate health care for migrant families with a newborn or young child. Both of these interventions were focused on child oral health.

The study by Gibbs et al. [24] was conducted in Melbourne, Australia, and was focused on families with one-to four-year old children from Iraqi, Lebanese or Pakistani migrant backgrounds. The intervention was a peer-led community oral health education program, which was delivered in culturally appropriate settings by peer educators from the same cultural and language background of the participants [24]. It sought to improve parent knowledge and behaviours relating to child oral health [24]. Gibbs et al. [24] found significantly less debris on the teeth of children in the intervention group compared to the control group. However, there were no other statistically significant differences between groups at follow-up in relation to other aspects of parent oral health knowledge [24]. A potential reason for the lack of significant outcomes was the low recruitment rate compounded by the high loss to follow up (47%), which meant that the study lacked power to detect differences.

The study by Harrison et al. [25] focused on Vietnamese migrant families with children under five. The intervention involved an adjunct to regular practice, with Vietnamese lay health counsellors providing one-on-one counselling to parents about their child’s oral health during regular immunisation visits to a Vietnamese Child Health Clinic. Harrison et al. [25] found that participating children had significantly fewer decayed surfaces than the comparison group, as well as significantly improved feeding practices compared to the comparison group. However, the follow-up rate was only 59% and it is possible that those who continued through to follow-up were more engaged in the intervention and more likely to have adopted healthy behaviours. Critical success factors mentioned in the study were that the lay health worker shared language, similar culture and refugee background with the study participants and was also a mother to young children [25]. Follow-up phone calls after the clinic visits helped to provide ongoing support and problem solve any difficulties [25]. Interacting with mothers when their children were very young was thought to be beneficial because it meant that the focus was not on changing existing behaviours, but rather, promoting and shaping adoption of helpful behaviours at the appropriate time [25]. Providing families with the necessary tools (e.g. a feeding cup was provided when it was time to wean the child from a bottle to the cup) to be able to adopt these behaviours was considered crucial, as a lot of the families had a low income [25].

## Discussion

The literature review illuminated a number of barriers and enablers to accessing health services for migrant parents with a newborn baby or young child. Plotting the barriers and enablers according to the different dimensions of the Access framework demonstrated that there are multiple points to intervene to improve access to care.

This review identified that the most significant barriers occurred on the supply side, within the ‘appropriateness’ domain. Interventions which address barriers to appropriateness such as poor communication may make the most difference to increasing access for migrant families. The most significant enablers also occurred on the supply side, but within the ‘acceptability’ domain. Making sure that these enablers are built into any interventions may also provide a way to maximise impact.

Similar findings were reported in a scoping review of health care access barriers for immigrants in Canada (not specific to parenthood), which found that the three most commonly reported barriers to accessing health care across 27 studies were 1) linguistic barriers, 2) lack of information about how to access or navigate services, and 3) cultural differences [26]. An Australian study exploring health professionals’ views on health literacy issues for culturally and linguistically diverse women in maternity care identified cultural barriers as a key issue, and also identified cultural awareness as an enabler [27]. The World Health Organization’s review of evidence on the reduction of inequalities in maternal health care delivery for migrants proposes incorporating indicators for culturally sensitive care into general indicators of good quality maternal care at health facilities [1].

Even though the studies in this review touched on concepts related to health literacy, such as language and culture, they did not discuss low health literacy specifically as being a barrier to access in this population group. However, it is known from the literature that people from diverse cultural and linguistic backgrounds and those who are experiencing socioeconomic disadvantage are more likely to have low health literacy [28] and be less engaged with self-management [29]. Low health literacy in patients is associated with poorer patient health outcomes, poorer medication adherence and poorer knowledge and understanding of their own condition. Patients with lower health literacy are less likely to use preventive services, are less likely to attend appointments and are more likely to be hospitalised [30, 31]. As such, further research into the influence of health literacy on access to postnatal care for migrant population groups would be beneficial, and may also be an avenue for intervention.

Our review process demonstrated that the majority of the literature on the topic of access to health services for migrant parents with a newborn baby/young child is qualitative, with a focus on describing issues rather than trialling interventions. This has also been noted by other authors, with Kalich et al. calling for more “solution-focused research … examining best practices and new policies and programming” [26]. We were only able to identify two studies which tested interventions. While the small number of intervention studies limited the conclusions that could be drawn, the studies did highlight a number of useful points for consideration. The importance of cultural humility and sensitivity was mentioned in the majority of the studies, and the use of bicultural/lay health workers was an integral part of the two intervention studies. Utilising peer workers enabled interventions to be delivered in a culturally appropriate manner and was also important for gaining the trust of participants. Social support was also frequently mentioned as an important factor in the studies, with social groups or multicultural mother’s groups mentioned as an enabler.

Some limitations of the review/evidence base are that only a small number of studies captured the father’s experience and only two studies were identified which evaluated an intervention (one of which was underpowered and both of which had high rates of drop-out). There appears to be considerably more research around the topic of prenatal care and the childbirth experience itself for migrant women than there is for the postnatal period. This may be because it is easier to engage people before they have a baby and when they are in hospital than it is once they have returned home. The study was also limited to interventions which occurred during the postnatal period. While outside of the scope of this review (which was focused on the postnatal period), some studies have examined the impact of antenatal interventions on access to care in the postnatal period. For example, Stapleton et al. [32] evaluated the introduction of a specialist antenatal clinic for women from refugee backgrounds. The authors found that while the clinic was valued by participants and ensured continuity of care throughout the antenatal period, participants reported a lack of continuity during labour and the postnatal period [32]. A qualitative study of new parents’ experiences of the transition to parenthood identified that while parents felt that the material provided in antenatal classes was useful, the material focused primarily on pregnancy and birth and they found that there was only limited discussion relating to the postnatal period [33]. A study comparing caseload midwifery care with standard midwifery care for women experiencing social disadvantage identified that women managed by caseload midwifery through the antenatal, intrapartum and postnatal period received greater numbers of referrals to multidisciplinary support services, although it is unclear whether these referrals included any postnatal support services [34]. Given that only a small number of postnatal interventions were identified in this review, future research exploring interventions across the full spectrum of antenatal, intrapartum and postnatal care would be beneficial.

## Conclusions

This review identified that there are a lack of interventions in the peer reviewed literature aiming to improve access to postnatal care for migrant families who speak a language other than English, with the majority of the literature taking a descriptive approach to identifying enablers and barriers to access to care. Despite this, a number of important considerations for practice emerged from this study. Firstly, the review highlighted the importance of utilising healthcare interpreters and cultural support workers during program delivery. Secondly, it demonstrates that staff attitudes and cultural understandings play a key role in people’s ability to engage with health services. Cultural humility is therefore an important aspect of health service training. Thirdly, the clustering of barriers on the ‘supply’ side of access to services suggests that interventions which focus on the health services rather than individuals may have the most impact on improving access and reducing health inequities for new migrant mothers.

Further trials and evaluation of programs should be conducted to add to the knowledge base about interventions that are effective. Working together with practitioners and consumers will be important to develop and refine program models. The findings of this review have been used to inform the development of a pilot intervention, co-designed with Child and Family Health Nurses and community members, to improve access to postnatal care for migrant mothers and their newborn children in Sydney Local Health District in Australia.

## Data Availability

All data generated or analysed during this study are included in this published article [and its supplementary information files].
